# Expanding the phenotype of TTLL5-associated retinal dystrophy: a case series

**DOI:** 10.1186/s13023-022-02295-9

**Published:** 2022-04-01

**Authors:** Jin Kyun Oh, José G. Vargas Del Valle, Jose Ronaldo Lima de Carvalho, Young Joo Sun, Sarah R. Levi, Joseph Ryu, Jing Yang, Takayuki Nagasaki, Andres Emanuelli, Nailyn Rasool, Rando Allikmets, Janet R. Sparrow, Natalio J. Izquierdo, Jacque L. Duncan, Vinit B. Mahajan, Stephen H. Tsang

**Affiliations:** 1 Department of Ophthalmology, Columbia University Irving Medical Center, New York, NY USA; 2 State University of New York at Downstate Medical Center, Brooklyn, NY USA; 3 School of Medicine, Medical Sciences Campus, University of Puerto Rico, San Juan, PR USA; 4 Department of Ophthalmology, Hospital das Clinicas de Pernambuco (HCPE) - Empresa Brasileira de Servicos Hospitalares (EBSERH), Federal University of Pernambuco (UFPE), Recife, Pernambuco Brazil; 5 Department of Ophthalmology, Federal University of São Paulo (UNIFESP), São Paulo, São Paulo Brazil; 6 Omics Laboratory, Byers Eye Institute, Stanford University, Palo Alto, CA USA; 7 Department of Ophthalmology, Medical Sciences Campus, University of Puerto Rico, San Juan, PR USA; 8 Department of Ophthalmology, University of California, San Francisco, San Francisco, CA USA; 9 Department of Pathology & Cell Biology, Columbia University Medical Center, New York, NY USA; 10 Department of Surgery, Medical Sciences Campus, University of Puerto Rico, San Juan, PR USA; 11 Veterans Affairs Palo Alto Health Care System, Palo Alto, CA USA; 12 Harkness Eye Institute, Columbia University Medical Center, 635 West 165th Street, Box 212, New York, NY 10032 USA

**Keywords:** TTLL5, Inherited retinal dystrophy, Retinitis pigmentosa, Cone–rod dystrophy, Cone dystrophy, Autosomal recessive

## Abstract

**Background:**

Inherited retinal dystrophies describe a heterogeneous group of retinal diseases that lead to the irreversible degeneration of rod and cone photoreceptors and eventual blindness. Recessive loss-of-function mutations in Tubulin Tyrosine Ligase Like 5 (*TTLL5)* represent a recently described cause of inherited cone–rod and cone dystrophy. This study describes the unusual phenotypes of three patients with autosomal recessive mutations in *TTLL5*. Examination of these patients included funduscopic evaluation, spectral-domain optical coherence tomography, short-wavelength autofluorescence, and full-field electroretinography (ffERG). Genetic diagnoses were confirmed using whole exome capture. Protein modeling of the identified variants was performed to explore potential genotype–phenotype correlations.

**Results:**

Genetic testing revealed five novel variants in *TTLL5* in three unrelated patients with retinal dystrophy. Clinical imaging demonstrated features of sectoral cone–rod dystrophy and cone dystrophy, with phenotypic variability seen across all three patients. One patient also developed high-frequency hearing loss during a similar time period as the onset of retinal disease, potentially suggestive of a syndromic disorder. Retinal structure findings were corroborated with functional measures including ffERG findings that supported these diagnoses. Modeling of the five variants suggest that they cause different effects on protein function, providing a potential reason for genotype–phenotype correlation in these patients.

**Conclusions:**

The authors report retinal phenotypic findings in three unrelated patients with novel mutations causing autosomal recessive *TTLL5*-mediated retinal dystrophy. These findings broaden the understanding of the phenotypes associated with *TTLL5*-mediated retinal disease and suggest that mutations in *TTLL5* should be considered as a potential cause of sectoral retinal dystrophy in addition to cone–rod and cone dystrophies.

**Supplementary Information:**

The online version contains supplementary material available at 10.1186/s13023-022-02295-9.

## Background

Tubulin Tyrosine Ligase Like 5 (*TTLL5*, OMIM #612268) is a gene that encodes a homonymous multifunctional protein involved in the post-translational polyglutamylation of α-tubulin [1–4]. *TTLL5* (NM_015072.5) has six isoforms of variable expression, but isoform 001 is the most prominent, with high levels of expression in the testes and retina, followed by the heart [1–4]. In vitro, *TTLL5* mutations were first described to cause decreased fertility in male mice secondary to disrupted axonemes in the sperm [4]. However, other α-tubulin dependent organs including the retina and cochlea were reportedly unaffected, contrary to disease caused by mutations in other ciliary genes, such as *BBS4*, which affect both the retina and spermatozoa [4, 5]. In humans, *TTLL5* mutations were first characterized as a cause of cone–rod dystrophy (CORD) or cone dystrophy (COD) and the protein was localized to the ciliary body of photoreceptors [6]. Within the retina, TTLL5 is responsible for the glutamylation of the ORF15 variant of the retinitis pigmentosa GTPase regulator (*RPGR*) gene, as both RPGR^ORF15^ and α-tubulin share similar homologous stretches of Glu-Gly repetitive regions [7]. Failed glutamylation of RPGR^ORF15^ may impair photoreceptor function and explain the retinal degeneration caused by mutations in *TTLL5*.

To date, nineteen cases of *TTLL5*-associated retinal dystrophy have been reported across sixteen different families [1, 6, 8, 9]. In all cases, disease was inherited in an autosomal recessive pattern, and the majority of patients were diagnosed with CORD. Phenotypic variability has been previously described, including a case that was initially diagnosed as atypical incomplete congenital stationary night blindness [8]. This report describes three patients with *TTLL5* mutations who presented with individually different phenotypes.

## Results

### Patient summary

**Fig. 1:**
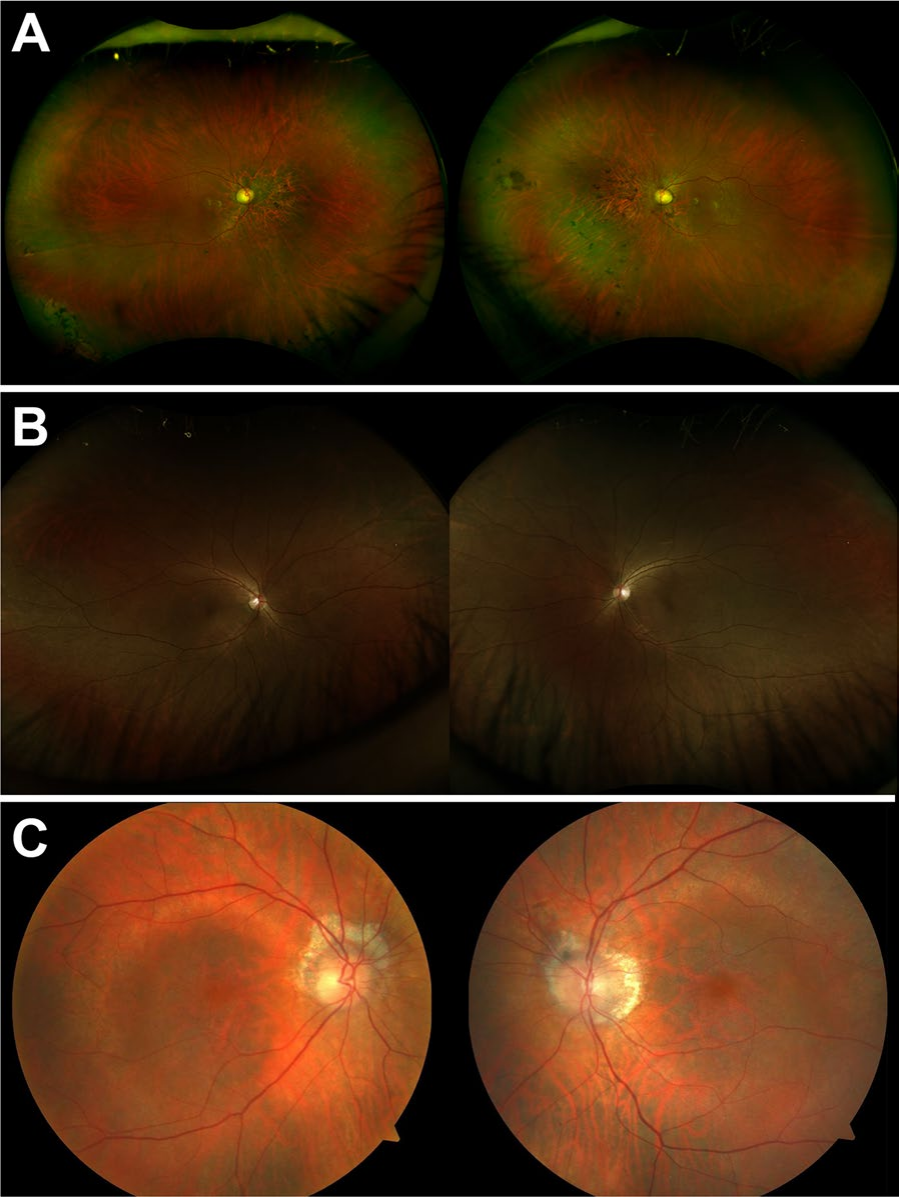
Short-wavelength fundus autofluorescence and wide-field color fundus photographs of three patients with mutations in *TTLL5*. P1 presented with cone–rod dystrophy characterized by sectoral atrophy affecting the macula and inferonasal retina with intraretinal pigment migration and exposure of the deep choroidal vessels (**A**). P2 exhibited a small central bull’s eye lesion and RPE mottling more pronounced in the right eye than the left (**B**). The fundus of P3 revealed prominent peripapillary and central macular atrophy with visualization of the choroidal vessels (**C**)

**Fig. 2:**
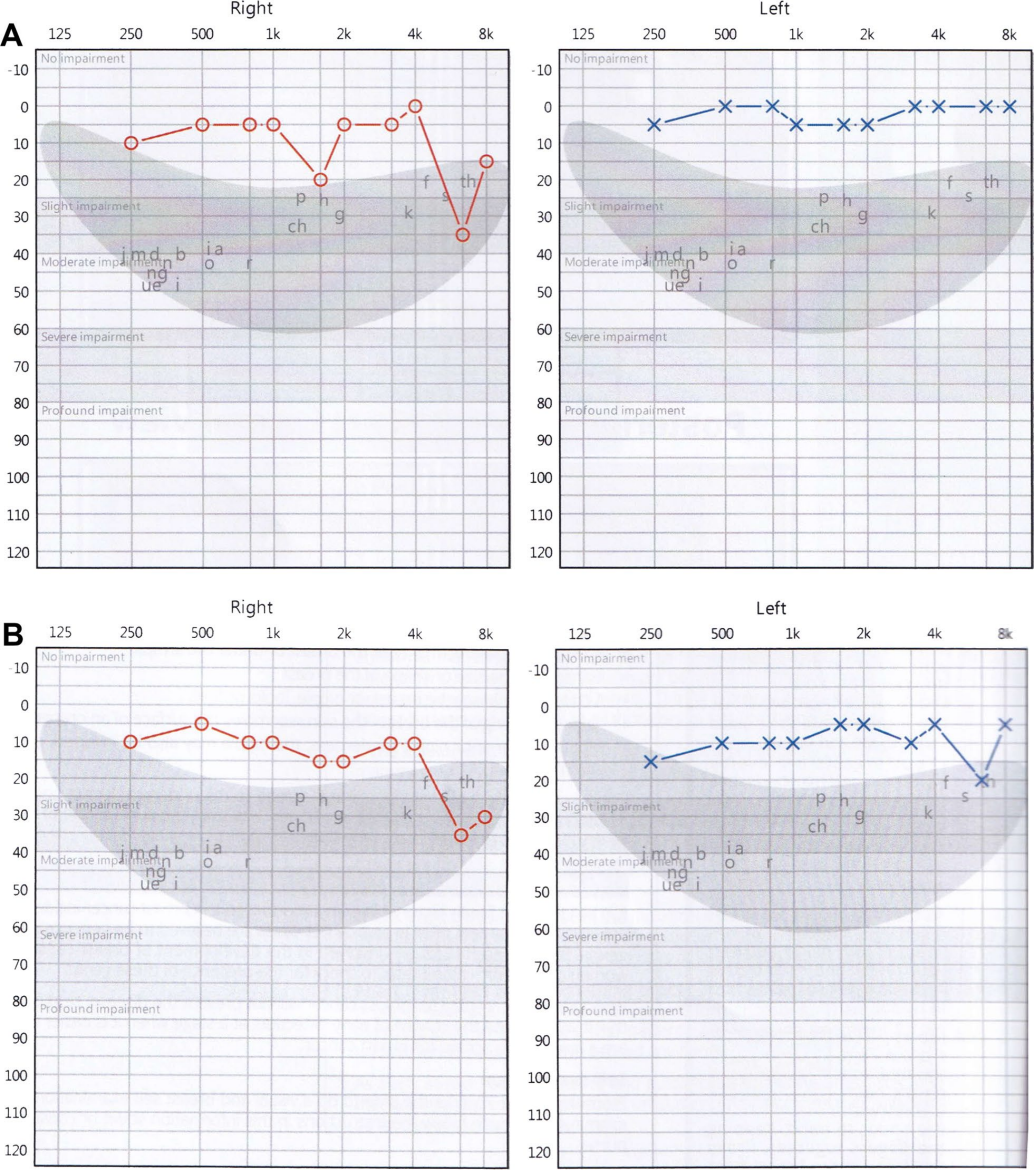
Audiograms over 2 years in P2. P2 presented with a history of high-frequency hearing loss at age 39 (**A**) only in the right ear at frequencies between 4000 and 8000 Hz. At age 40 (**B**), slight impairment was also appreciated at the same frequencies in the left ear

Table 1 summarizes the clinical, genetic, and demographic information of all three patients (P1-P3). Two patients (P1 and P2) initially presented with nyctalopia and photophobia (P1) or dyschromatopsia (P2) while P3 presented with complaints of persistent blurry vision that did not improve with refraction. Dilated fundus examination revealed peripapillary atrophy and vascular attenuation in P1 and P3, with atrophy and exposure of the deep choroidal vessels in the macula (P3) and in a sectoral distribution nasal to the optic disc (P1) (Fig. 1A, C). The fundus of P2 showed a central bull’s eye lesion and RPE mottling (Fig. 1B). P2 also endorsed a 2-year history of high-frequency hearing loss demonstrated in sequential audiograms, which demonstrated slight bilateral impairment at higher frequencies with sparing at lower frequencies (Fig. 2).

**Table 1:** Patient demographics, genotypes, and phenotypic findings

Patient ID	Gender	Ethnicity	Age at diagnosis (years)	Age at most recent evaluation (years)	Variants and ACMG classification	BCVA (OD, OS)	Phenotype
P1	M	Caucasian	50	70	c.1450C>T: p.(Arg484Cys) [VOUS] c.2987del: p.(Gly996Aspfs*) [Likely pathogenic]	20/250, 20/300	Sectoral cone–rod dystrophy
P2	F	South Chinese	37	41	c.1475G>A: p.(Trp492*) [Pathogenic] c.3177_3180del: p.(Asn1060*) [Pathogenic]	20/30, 20/30	Cone dystrophy and hearing loss
P3	F	Puerto Rican	46	50	c.2029C>T: (p.Arg677*) homozygous [Pathogenic]	20/400, 20/400	Cone–rod dystrophy

*M* male, *F* female, *ACMG* American College of Medical Genetics and Genomics, *VOUS* variant of unknown significance, *BCVA* best corrected visual acuity, *OD* right eye, *OS* left eye

### Retinal imaging

**Fig. 3:**
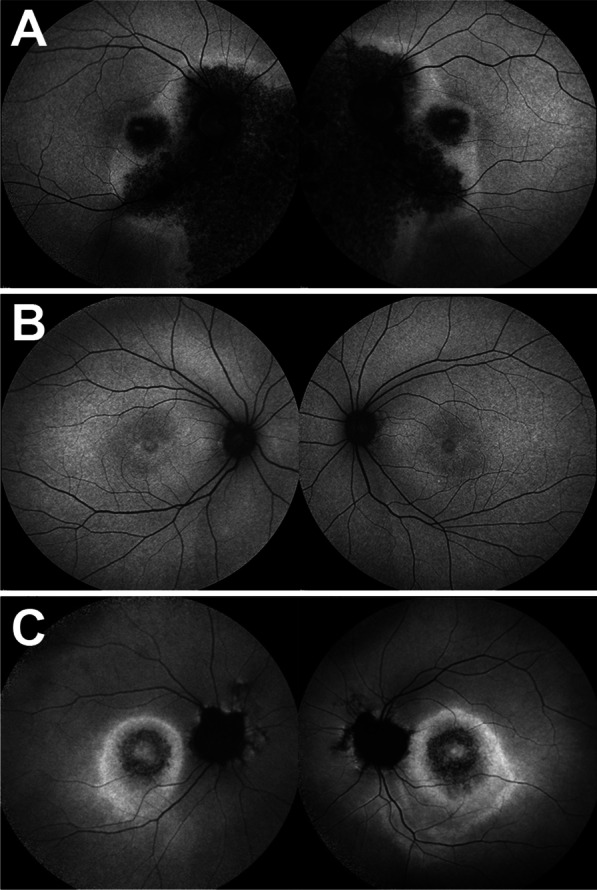
Short-wavelength fundus autofluorescence of three patients with mutations in *TTLL5*. Fundus autofluorescence images of P1 revealed sectoral atrophy involving the macula and the inferonasal retina with an irregular hyperautofluorescent ring surrounding the areas of atrophy (**A**). P2 exhibited a bull’s eye pattern of foveal hypoautofluorescence, more pronounced in the left than in the right eye (**B**). The foveal hypoautofluorescence secondary to macular pigment usually observed in normal eyes was not distinctly evident. P3 demonstrated a central bull’s eye pattern of hypoautofluorescence surrounded by a larger hyperautofluorescent ring extending further inferiorly than superiorly (**C**)

**Fig. 4:**
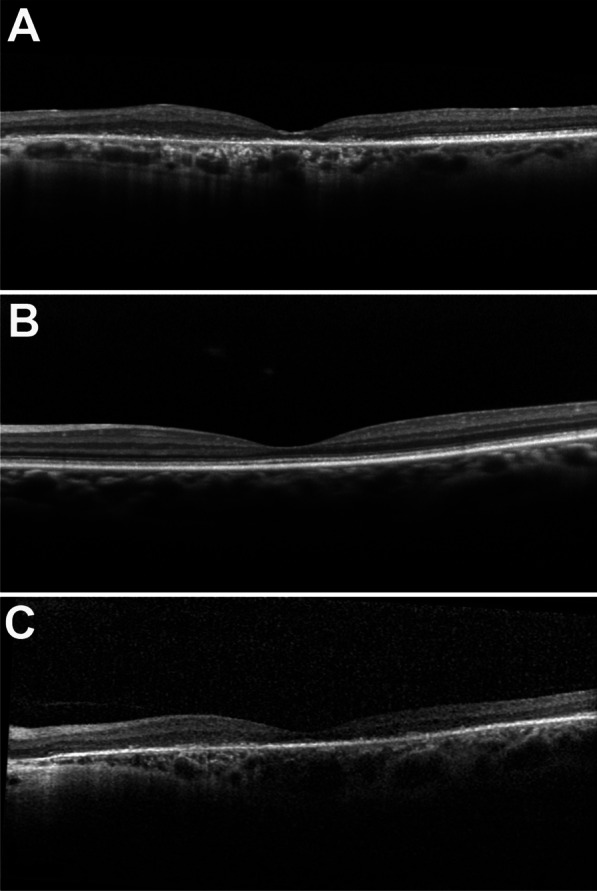
Spectral-domain optical coherence tomography scans in three patients with mutations in *TTLL5*. Spectral-domain optical coherence tomography of P1 revealed central macular atrophy and diffuse retinal thinning consistent with cone–rod dystrophy (**A**). P2 demonstrated relatively preserved retinal architecture with the exception of disruption of the ellipsoid zone band in the distribution of the hypoautofluorescent ring around the fovea and thinning of the outer nuclear layers (**B**). P3 was found to have diffuse macular atrophy with retinal thinning and RPE atrophy (**C**). A small area of spared ellipsoid zone can be observed along the temporal border of the macula

In short-wavelength autofluorescence images (SW-AF), all three patients presented with different phenotypes as seen in Fig. 3. P1 presented with sectoral atrophy affecting the macula and extending along the inferotemporal retinal vein and nasal to the optic disc (Fig. 3A). Spectral-domain optical coherence tomography (SD-OCT) revealed central macular atrophy and retinal thinning more pronounced in the nasal retina, with RPE atrophy suggested by hypertransmission into the choroid (Fig. 4A). SW-AF images of P2 showed a bull’s eye pattern with a ring of hypoautofluorescence surrounding the fovea (Fig. 3B), more pronounced in the left than the right eye. The ellipsoid zone band was irregular on SD-OCT in P2 in a region similar to the hypoautofluorescent ring visible in SW-AF images, with thinning of the outer nuclear layer at the fovea (Fig. 4B). P3 demonstrated a bull’s eye pattern of macular atrophy on SW-AF with a hyperautofluorescent ring surrounding central hypoautofluorescence (Fig. 3C). SD-OCT revealed diffuse macular atrophy and retinal thinning with RPE atrophy (Fig. 4C).

### Electroretinography

**Fig. 5:**
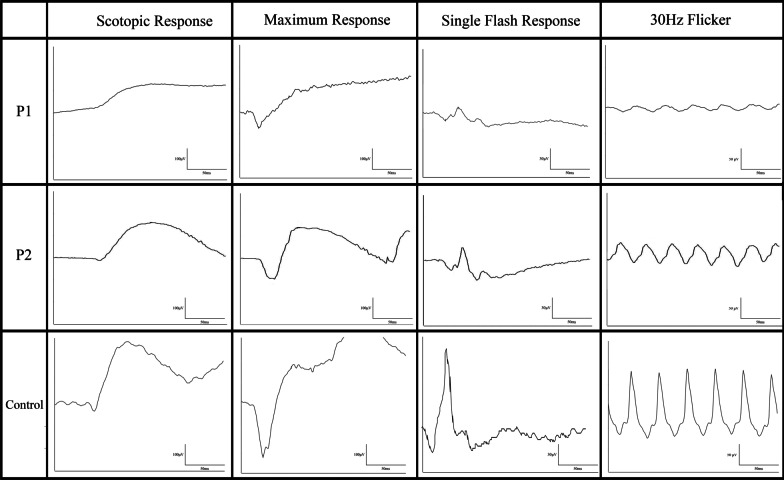
Full field electroretinogram findings of *TTLL5*. P1 demonstrated subnormal and delayed rod responses with decreased cone amplitudes and implicit time delay, consistent with a diagnosis of cone–rod dystrophy. P2 presented with normal rod response and cone response with decreased amplitude but no implicit time delay, consistent with cone dystrophy. A control patient was provided as reference

Full-field electroretinogram (ffERG) findings were consistent with the phenotypes observed in SW-AF images (Fig. 5). Table 2 summarizes ffERG amplitudes and timing for P1 and P2. P1 showed subnormal scotopic responses and 30 Hz photopic flicker amplitudes with implicit time delays, consistent with a diffuse cone greater than rod pattern of dysfunction. P2 showed ffERG normal scotopic responses but diminished photopic single flash and 30 Hz flicker responses with preserved implicit timing. Multifocal-electroretinogram responses showed decreased amplitudes with no implicit time delays (Additional file 1: Fig. S1). The lack of diffuse outer retinal dysfunction with reduced macular responses were consistent with the diagnosis of COD.

**Table 2:** Full-field electroretinogram findings

Patient ID	Scotopic response OD and OS (μV)	Scotopic response time OD and OS (ms)	Maximum response OD and OS A wave (μV)	Maximum response OD and OS B wave (μV)	Cone response OD and OS A wave (μV)	Cone response OD and OS B wave (μV)	30 Hz photopic flicker OD and OS (μV)	30 Hz flicker implicit time OD and OS (ms)
P1	113.7/115.1	111/108	− 104.3/− 75.2	154.3/141	10.6/12.6	17.9/11.2	19.3/13.6	35/36
P2	147.7/161.3	105/104	− 92/− 92.2	232.4/235.3	13.9/14.2	27.0/31.4	32.2/42.6	26/30

*OD* right eye, *OS* left eye

### Variant identification

**Fig. 6:**
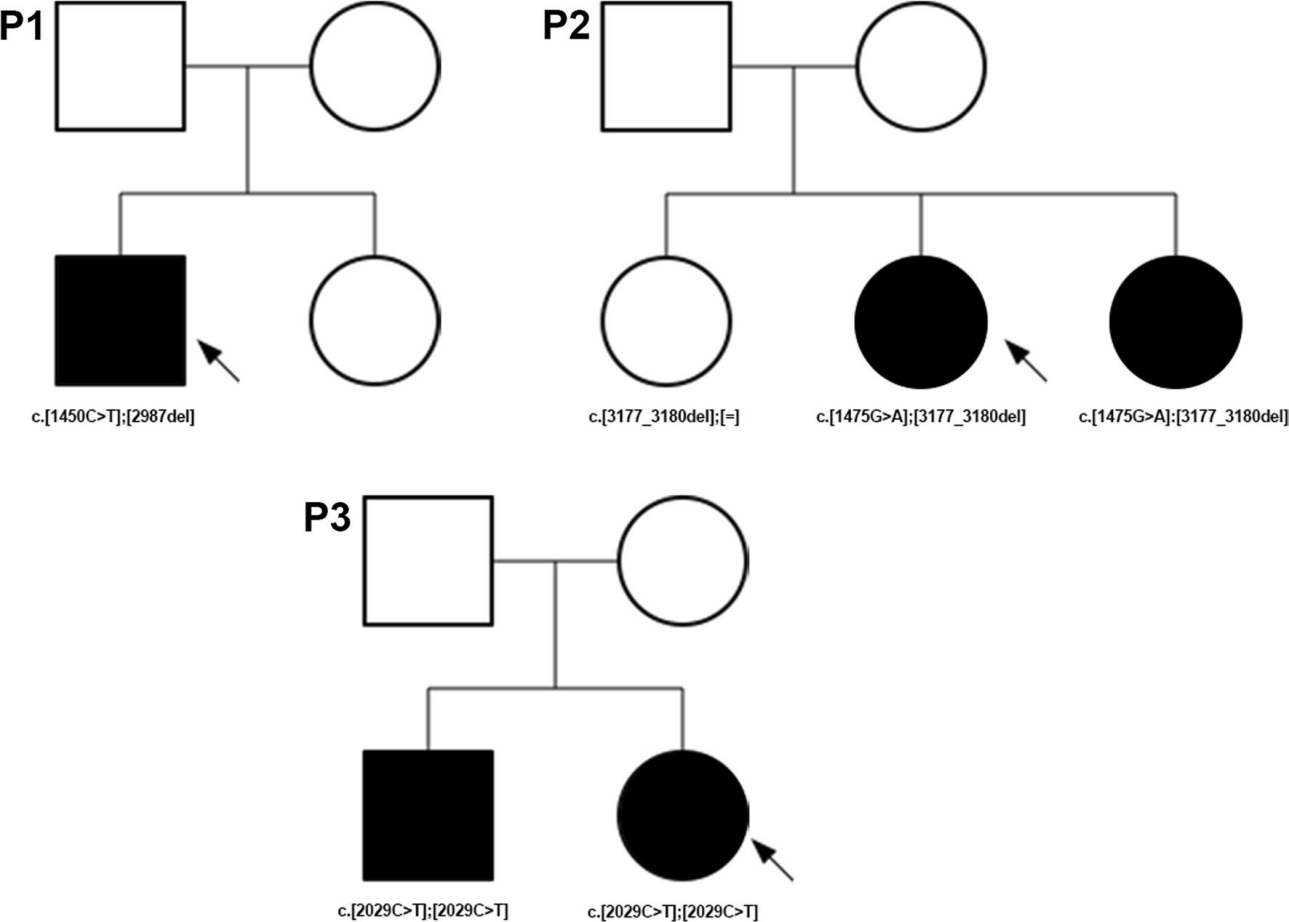
Pedigrees of three patients with mutations in *TTLL5*. P1 was found to possess two heterozygous mutations in *TTLL5* with segregation of variants that was performed in an unaffected sister and mother (**A**). The family declined to report which of the family members carried which variant; however, the performing laboratory confirmed that the mutations were located on opposing chromosomes. P2 was found to possess two heterozygous mutations in *TTLL5* with segregation of the variants performed in two asymptomatic sisters (**B**). The older sibling was found to be a heterozygous carrier of one variant while the younger sibling was found to carry both heterozygous mutations but had not been evaluated by an ophthalmologist at the time. P3 was found to possess a homozygous variant that was also identified in an affected brother (**C**). While segregation in unaffected family members was not performed, the laboratory ruled out any deletions that may be responsible for homozygosity

Two novel compound heterozygous variants in the *TTLL5* gene were present in P1, c.1450C>T:p.(Arg484Cys) and c.2987del:p.(Gly996Aspfs*5). The missense variant was classified as a variant of unknown significance and was predicted to be deleterious by all in silico prediction tools [SIFT: 0.014, Polyphen2: 1, MutationTaster: D, Provean: − 7.69, CADD Phred: 27.6], while the frameshift mutation was classified as likely pathogenic. The missense variant was found at a minor allele frequency of 0.0003 in population databases (gnomAD) while the frameshift variant was not found. Both variants occur at evolutionarily conserved residues and no homozygotes of either variant have been reported. Segregation analysis of the variants in the unaffected sister and mother confirmed that the variants were located on separate chromosomes (Fig. 6). Identification of which variants the unaffected family members carried was not reported by the laboratory at the request of the family.

P2 also showed two novel nonsense, stop-gained variants, c.1475G>A:p.(Trp492*) and c.3177_3180del:p.(Asn1060*), which were both classified as pathogenic and occur at evolutionarily conserved residues. Variant c.1475G>A:p.(Trp492*) has not been previously reported in gnomAD while variant c.3177_3180del:p.(Asn1060*) was found at a frequency of 0.0002 in gnomAD; however, two homozygotes with the second frameshift variant were found in population databases. Segregation analysis of the variants in two asymptomatic sisters illustrated that the older sibling carried a single heterozygous mutation, while the younger sibling possessed both mutations (Fig. 6). The younger sibling was asymptomatic but had not undergone full ophthalmic examination.

P3 was found to possess a homozygous frameshift mutation, c.2029C>T: (p.Arg677*), which was classified as a pathogenic variant and occurs at an evolutionarily conserved residue. The frameshift variant is found at a minor allele frequency of 0.00003 in gnomAD and no homozygotes of the variant have been previously reported. A similarly symptomatic sibling was found to also possess the same homozygous frameshift mutation (Fig. 6). While segregation of the variants was not performed in unaffected family members, homozygosity due to a large deletion was ruled out by the performing laboratory (Invitae laboratory, phone call, September 2021).

### Structural modeling

**Fig. 7:**
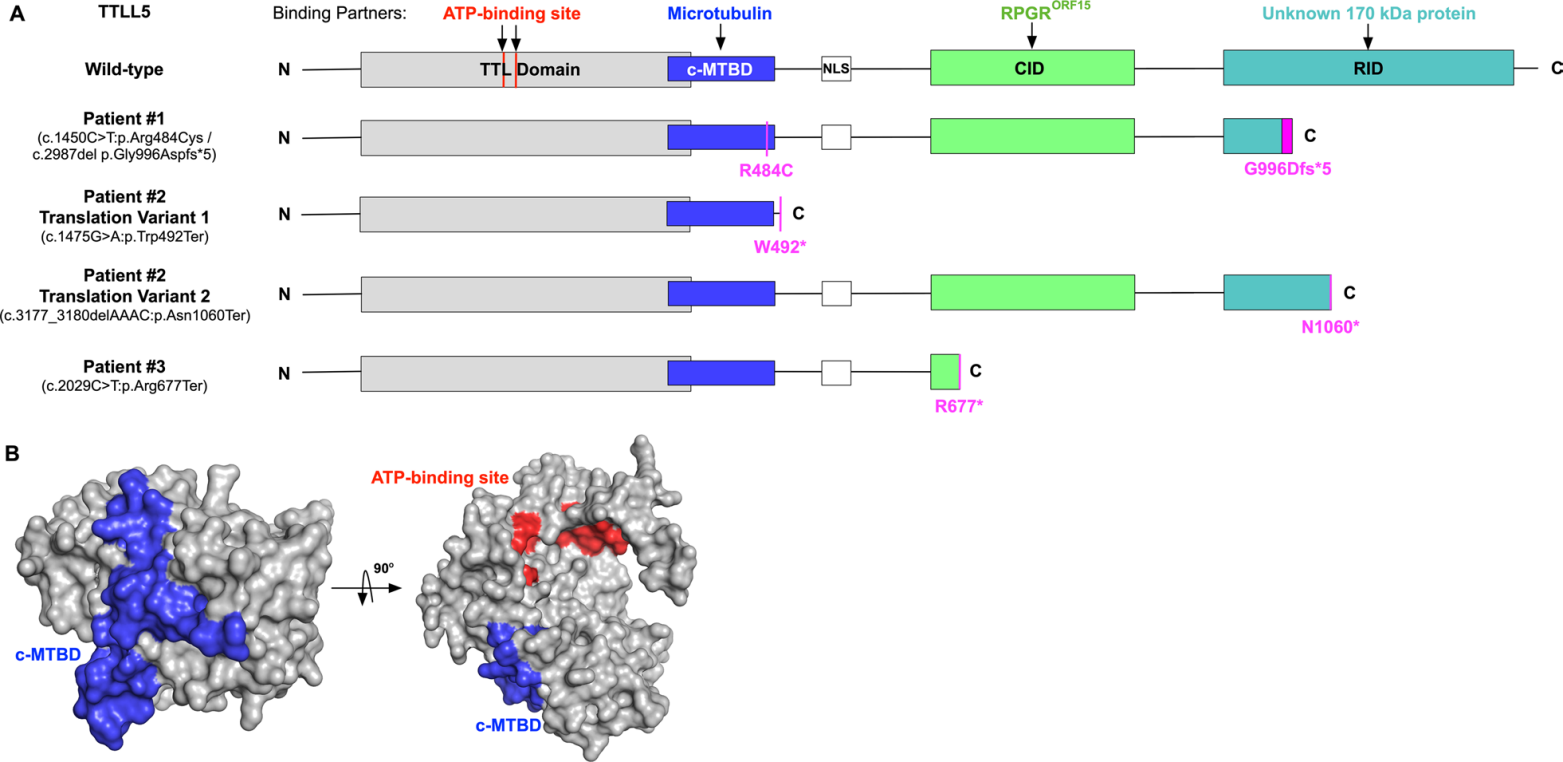
Structural modeling of TTLL5 domain shows mutants disrupt interactions with binding partners. The domain topology of wild-type TTLL5 and patient mutants are illustrated (**A**). The TTL domain is shown in the grey box, the c-MTBD is shown in the blue box, the nuclear localization signal region is shown in the white box, the CID is shown in the green box, and the RID is shown in the magenta box. The ligands and their corresponding binding domains are indicated with arrows. Each patient mutation is shown in magenta. The homology-based model of the TTLL5 TTL domain is demonstrated in a surface model (**B**). The ATP-binding site is highlighted in red, and the microtubulin-binding site is highlighted in blue

The TTLL5 protein contains four domains: a tubulin-tyrosine ligase domain (TTL), a c-terminal microtubule binding domain (c-MTBD), a cofactor interaction domain (CID), and a receptor interaction domain (RID) [4, 8]. The TTL domain is an enzymatic domain that glutamylates ligands with the help of adenosine triphosphate (ATP) [4]. The other three domains are ligand recognition domains: c-MTBD interacts with microtubulins, CID interacts with the ORF15 region of RPGR^ORF15^, and RID interacts with an unknown protein that is approximately 170 kDa [4, 8]. Figure 7 shows the locations of the identified variants in patients P1-P3 relative to the functional domains of the protein.

The c.1450C>T:p.(Arg484Cys) variant in P1 is located within the c-MTBD. It reduces microtubule binding by causing the loss of a positively charged arginine, which could interact with the negatively charged c-terminal of ß-tubulin. In contrast, the c.2987del:p.(Gly996Aspfs*5) variant causes the insertion of a premature stop codon leading either to the truncation of the protein or to the decay of the resulting transcript [10]. In P2, both the c.1475G>A:p.(Trp492*) variant and the c.3177_3180del:p.(Asn1060*) variant introduce a premature stop codon that would lead to shortening of the protein prior to the CID or RID respectively, or to degradation by nonsense mediated decay. Similarly, the c.2029C>T:p.(Arg677*) variant identified in P3 causes the insertion of a stop codon in the CID, leading to truncation of the CID or to the decay of the transcript.

## Discussion

TTLL5 is a multifunctional protein involved in polyglutamylation of α-tubulin and modulation of glucocorticoid receptors [2–4]. *TTLL5*-mediated disease was first described in 2014 to cause isolated CORD, however, rare reports of unusual phenotypes have been reported [6, 8]. In this study, three patients with recessive mutations in *TTLL5* showed three unique phenotypes: sectoral CORD, CORD, and COD. The age of onset among these three patients was variable, as were presenting symptoms, suggesting that *TTLL5-*mediated retinal dystrophy may be more phenotypically varied than previously reported [1, 6, 8, 9].

It has been suggested that truncating mutations in *TTLL5* may cause syndromic disease with azoospermia in addition to retinal disease, while missense mutations lead to isolated retinal findings [1]. A patient with *TTLL5* mutations has been reported with azoospermia, similar to mice with *TTLL5*-deficiency [7]. However, three other male patients with truncating mutations in *TTLL5* who had produced offspring have been reported [6]. In our study, one female patient (P2) had a truncating mutation in *TTLL5*. While azoospermia was not a concern for this patient, the patient had a history of high-frequency hearing loss supported by audiograms taken over 2 years. Other ciliopathies that affect hearing, such as Usher Syndrome, also cause more severe hearing loss at higher than lower frequencies [11]. Two other patients with homozygous truncating mutations in *TTLL5* (c.1627G>T:p.(Glu543*) and c.1782del:p.(Asp594Glufs*29)), also had bilateral mixed hearing loss; neither of them had children, and one was noted to have azoospermia after semen analysis [1, 6]. The reported expression of *TTLL5* within the inner ear of mice and the high frequency hearing loss seen in P2 and previously reported patients raise the possibility that truncating mutations in *TTLL5* may cause syndromic disease that includes azoospermia, retinal findings, and high-frequency hearing loss (David He, MD, PhD, email communication, May 2020) [12–16]. No other variants in genes responsible for hearing loss were identified. Given that only a small number of patients with truncating mutations in *TTLL5* have been reported, a larger cohort should be evaluated to elucidate syndromic phenotypes. Further study is also necessary to validate the expression of *TTLL5* within the inner ear of humans.

Structural modeling and protein interactions within the four domains of TTLL5 suggest a possible explanation for the three distinct phenotypes seen in our patients. The c.2987del:p.(Gly996Aspfs*5) variant found in P1 was predicted to cause the loss of interaction between the unknown 170 kDa protein and the RID, which has a similar impact on the protein as the previously described c.3354G>A:p.(Trp1118*) variant [6, 7]. However, P1 presented at a later age and was found to have peripheral sectoral atrophy seen on SW-AF, while a previously reported patient with the c.3354G>A:p.(Trp1118*) variant presented at a young age and was found to have a hyperautofluorescent ring surrounding central macular atrophy on SW-AF. In contrast, the c.1450C>T:p.(Arg484Cys) missense variant identified in P1 occurs within close proximity of the c.1474T>A:p.(Trp492Arg) missense variant that was recently reported in a study by Smirnov et al. [9] The patient who possessed this variant was found to have sectoral atrophy on examination with an onset of symptoms in his mid-forties, similarly to P1, suggesting that this area within the c-MTBD may have a genotype–phenotype correlation with sectoral atrophy.

P2 was found to have two different stop-gain variants. Typically, stop-gain variants have been associated with CORD, but cases with mildly affected cone response have also been described [1, 6]. The lack of 30 Hz flicker photopic implicit time delay in P2 suggests that the cone photoreceptors are not diffusely affected. P3 was similarly found to have a homozygous stop-gain variant but was found to have a significantly more advanced phenotype. The reason for the difference in phenotype severity between these patients is unclear, especially given that nonsense mutations are expected to lead to loss of function secondary to nonsense mediated decay [11]. However, the homozygous stop-gain variant found in P3, c.2029C>T:p.(Arg677*), is located in close proximity to the homozygous stop-gain variant c.1920G>A:p.(Trp640*) identified in a recently described patient diagnosed with a more severe early onset phenotype, both of which occur within the CID [9]. These findings suggest that mutations located around this locus may be associated with more advanced disease. Taken together, these findings suggest that genotype–phenotype correlation may exist in *TTLL5*, and further studies assessing larger cohorts with be valuable in confirming the presence of domain-specific or mutation type-dependent correlations.

## Conclusions

As the current understanding and treatment of retinal dystrophies is gene-specific, the characterization and genotype–phenotype correlation of variants is important for future management. In this study, the phenotype of *TTLL5*-related retinal degeneration is expanded to include mild COD and sectoral CORD, and structural modeling was performed to elucidate potential mechanisms of disease pathogenesis. *TTLL5* mutations should be considered in the differential diagnoses for sectoral retinal dystrophies and mild COD. Further characterization of patients with *TTLL5-*related retinal degeneration is required and will ultimately be beneficial in the future treatment of this condition, both in understanding the natural history of disease progression and identifying suitable outcome measurements for therapy.

## Methods

### Subjects

Three patients from three unrelated families were seen and evaluated at Columbia University Irving Medical Center, the University of California, San Francisco, and the University of Puerto Rico. Informed consent was waived due to the minimal risk conferred to the patients and the retrospective nature of the study design as per the Institutional Review Board at Columbia University (protocol AAAR8743), the University of California, San Francisco (protocol 21-33974), and the University of Puerto Rico (protocol B1960120), and all procedures were reviewed and in accordance with the tenets of the Declaration of Helsinki. The data presented in this study was procured through retrospective chart review. Diagnosis was made based on clinical evaluation and supported by genetic testing.

### Examination

Ophthalmic examination involved measurement of best corrected visual acuity and pupil dilation with phenylephrine (2.5%) and tropicamide (1%). Dilation was followed by fundus examination, photography, SD-OCT, and SW-AF (488 nm excitation, barrier filter transmitted light from 500 to 680 nm, 55° × 55° field autofluorescence). SD-OCT and SW-AF were acquired using a Spectralis HRA + OCT (Heidelberg Engineering, Heidelberg, Germany). Digital color fundus and ultrawide-field color fundus photographs (Optos 200 Tx, Optos PLC, Dunfermline, United Kingdom) were also obtained.

### Electroretinography

ffERG was performed using DTL electrodes and Ganzfeld stimulation on a Diagnosys Espion Electrophysiology System (Diagnosys LLC, Littleton, MA, USA) according to international standards [17].

### Whole exome sequencing and variant analysis

DNA was isolated from the peripheral blood of each patient for analysis. P1 underwent whole exome sequencing (WES) at the clinical laboratory improvement amendments (CLIA)-approved Laboratory of Personalized Genomic Medicine at Columbia University Medical Center (New York, NY). P2 underwent WES at the CLIA-approved Molecular Vision Laboratory (Hillsboro, OR). WES was performed using Agilent SureSelectXT Human All Exon V5 + UTRs (Agilent Technologies, Santa Clara, CA) and Illumina HiSeq X (Illumina, San Diego, CA). Familial samples of both P1 and P2 were sequenced in order to confirm the phase of the identified variants. P3 underwent panel testing performed through the CLIA-approved Invitae laboratory (San Francisco, CA). Analysis for deletions and duplications was performed by Invitae using an internal algorithm to compare read-depth for each target in the proband sequence with both read-depth distribution and mean read-depth from a larger set of clinical samples. The absence of a large deletion was specifically re-assessed in P3 to rule out potential homozygosity due to the presence of a large deletion (Invitae laboratory, phone call, September 2021).

Was re-assessed in P3 to rule out potential homozygosity due to the presence of a large deletion. All suspected disease-associated variants were confirmed through Sanger sequencing and classified according to ACMG guidelines [18]. The variants’ predicted effects were determined using in silico prediction software including SIFT, Polyphen-2, Mutation Taster, Provean, and CADD Phred.

### Structural modeling of TTLL5

The online PHYRE2 server was used to generate the homology-based structural model of TTLL5 [19, 20]. The tubulin-tyrosine ligase domain structure of TTLL5 (residues from 65 to 417) was modeled with high confidence based on the TTL domain structure of TTLL7 (PDB ID: 4YLR). The N-terminal half of c-terminal microtubule binding domain was modeled as a part of TTL domain. The C-terminal half of c-MTBD was not modeled, because it is typically disordered and gains α-helical secondary structure only upon microtubulin binding [16]. There were no known structures to model the cofactor interaction domain and the receptor interaction domain structure of TTLL5 in high confidence. The figure was generated using Pymol (The PyMOL Molecular Graphics System, Version 2.0 Schrödinger, LLC).

## Supplementary Information

**Additional file 1: Fig. S1.** Multifocal electroretinogram findings of P2.P2 underwent multifocal electroretinogram testing which demonstrated reduced amplitudes across all six rings in both eyes. Despite the amplitude reduction, implicit times were normal, consistent with a mild cone dystrophy.

## Data Availability

The datasets used and/or analysed during the current study are available from the corresponding author on reasonable request.

## Abbreviations

SD-OCT: Spectral-domain optical coherence tomography
SW-AF: Short wavelength autofluorescence
RPE: Retinal pigment epithelium
ff-ERG: Full-field electroretinogram
DTL: Dawson, Trick, Litzkow
TTL: Tubulin-tyrosine ligase
c-MTBD: c-Terminal microtubule binding domain
CID: Cofactor interaction domain
RID: Receptor interaction domain
CLIA: Clinical Laboratory Improvement Amendments
WES: Whole exome sequencing
CORD: Cone–rod dystrophy
COD: Cone dystrophy
