# Supplementary figures and images for: Expanding the phenotype of TTLL5-associated retinal dystrophy: a case series
Source: Orphanet J Rare Dis. 2022 Apr 1;17:146. doi: 10.1186/s13023-022-02295-9 (PMC8973795; doi:10.1186/s13023-022-02295-9)

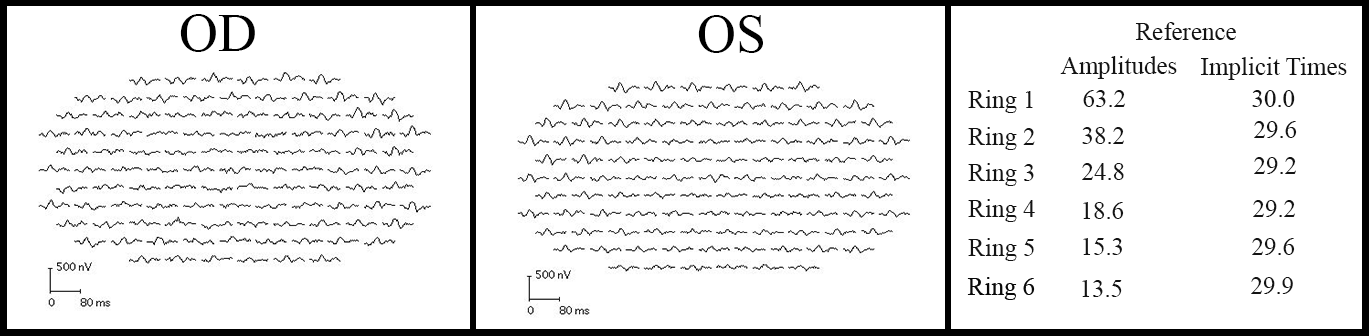

Supplement: Supplementary file 1 — Additional file 1: Fig. S1. Multifocal electroretinogram findings of P2.P2 underwent multifocal electroretinogram testing which demonstrated reduced amplitudes across all six rings in both eyes. Despite the amplitude reduction, implicit times were normal, consistent with a mild cone dystrophy. [file 13023_2022_2295_MOESM1_ESM.png]
